# The Nucleosome-Remodeling ATPase ISWI Is Regulated by Poly-ADP-Ribosylation

**DOI:** 10.1371/journal.pbio.0060252

**Published:** 2008-10-14

**Authors:** Anna Sala, Gaspare La Rocca, Giosalba Burgio, Elena Kotova, Dario Di Gesù, Marianna Collesano, Antonia M. R Ingrassia, Alexei V Tulin, Davide F. V Corona

**Affiliations:** 1 Istituto Telethon Dulbecco, Universita' degli Studi di Palermo, Palermo, Italy; 2 Dipartimento di Scienze Biochimiche, Universita' degli Studi di Palermo, Palermo, Italy; 3 Fox Chase Cancer Center, Philadelphia, Pennsylvania, United States of America; University of California, San Diego, United States of America

## Abstract

ATP-dependent nucleosome-remodeling enzymes and covalent modifiers of chromatin set the functional state of chromatin. However, how these enzymatic activities are coordinated in the nucleus is largely unknown. We found that the evolutionary conserved nucleosome-remodeling ATPase ISWI and the poly-ADP-ribose polymerase PARP genetically interact. We present evidence showing that ISWI is target of poly-ADP-ribosylation. Poly-ADP-ribosylation counteracts ISWI function in vitro and in vivo. Our work suggests that ISWI is a physiological target of PARP and that poly-ADP-ribosylation can be a new, important post-translational modification regulating the activity of ATP-dependent nucleosome remodelers.

## Author Summary

The ISWI protein is a highly conserved nucleosome remodeler that plays essential roles in regulating chromosome structure, DNA replication, and gene expression. The variety of functions associated with ISWI activity are probably connected to the ability of other cellular factors to regulate its ATP-dependent nucleosome-remodeling activity. We identified one factor—the poly-ADP-ribose polymerase, PARP—that can counteract ISWI function. PARP is an abundant nuclear protein that catalyzes the transfer of ADP-ribose units to specific proteins involved in DNA repair, transcription, and chromatin structure. Our work suggests that the activity of an ATP-dependent remodeler can be modulated by poly-ADP-ribosylation in order to regulate chromatin function in vivo.

## Abstract 3

Enzymes that mediate nucleosome remodeling and poly-ADP-ribosylation play essential roles in the eukaryotic cell. A new study suggests a mechanism to explain how two nuclear enzymes can coordinate their activities to regulate chromatin structure and function.

## Introduction

Eukaryotic chromatin is packaged in a highly organized hierarchy of structural building blocks, all composed of the basic repeating unit of the nucleosome. ATP-dependent nucleosome-remodeling activities as well as covalent modifications of chromatin components underlie the dynamic nature of chromatin structure and function [1,2]. Although it is expected that a crosstalk should exist between ATP-dependent remodelers and covalent modifiers of chromatin, very little is known about how these activities are integrated and coordinated with each other.

ISWI is the catalytic subunit of several ATP-dependent nucleosome remodeling complexes. ISWI is highly conserved during evolution and is essential for cell viability [3]. ISWI-containing complexes are thought to play central roles in DNA replication, gene expression, and chromosome organization [4]. ISWI uses the energy of ATP hydrolysis to catalyze nucleosome spacing and sliding reactions [3]. In *Drosophila*, loss of ISWI function causes global transcription defects and leads to dramatic alterations in higher-order chromatin structure, including the apparent decondensation of both mitotic and interphase chromosomes [5,6]. Recent findings indicate that ISWI controls chromosome compaction in vivo, in part through its ability to promote chromatin association with the linker histone H1 [5].

In vitro and in vivo studies carried out in several model organisms have also shown the involvement of ISWI complexes in a variety of nuclear functions including telomere silencing, stem cell self-renewal, neural morphogenesis, and the epigenetic reprogramming that occurs during nuclear transfer in animal cloning [4,7,8]. Remarkably, inactivation of ISWI interferes with the Ras pathway [9], and loss of ISWI function seems to be associated with a subset of melanotic tumors and the human multi-systemic disease Williams-Beuren syndrome [10].

The variety of functions associated with ISWI activity are probably connected to the ability of other cellular factors to regulate its ATP-dependent chromatin remodeling activity. Indeed, nucleosome spacing reactions catalyzed by ISWI can be regulated by its associated subunits [11]. However, evidences in vitro and in vivo indicate that ISWI activity can also be directly regulated by acetylation [12] and site-specific acetylation of histones [13]. We recently found that ISWI function could be modulated in vivo by a variety of cellular factors that escaped previous biochemical analyses. Indeed, in an unbiased genetic screen for factors modifying phenotypes caused by loss of *ISWI* function, we identified new potential regulators of ISWI in the higher eukaryote Drosophila melanogaster [14].

One class of mutants isolated in the screen is made up of chromatin components and nuclear enzymatic activities that could regulate ISWI function by covalently modifying histones or ISWI itself. In this class we found mutants in the gene encoding for the poly-ADP-ribose polymerase—*Parp*—and the gene encoding for poly-ADP-ribose glycohydrolase—*Parg*—[14], two conserved nuclear enzymes that catalyze the transfer and the removal, respectively, of ADP-ribose units to a wide variety of target proteins, using NAD^+^ as a substrate, to regulate chromatin accessibility in *Drosophila* [15]. The activities of PARP and PARG have been implicated in modulating chromatin structure, gene expression and the response to DNA damage [16–18].

The genetic interactions identified between *ISWI*, *Parp*, and *Parg* suggest that poly-ADP-ribosylation reactions could be coordinated and integrated within the activity of the ATP-dependent chromatin-remodeling factor ISWI. Here we present data showing that the nucleosome remodeling factor ISWI is poly-ADP-ribosylated in vitro and in vivo*.* The poly-ADP-ribosylation of ISWI inhibits its ATPase activity by reducing the affinity of ISWI with its nucleosomal substrate. We found that ISWI and PARP bind different chromatin domains and that the in vivo induction of chromatin poly-ADP-ribosylation results in loss of ISWI chromatin binding, suggesting that poly-ADP-ribosylation of ISWI might favor its dissociation from chromatin. One of the central questions in the study of PARP biology is the functional role played by the poly-ADP-ribose as a covalent epigenetic mark [22]. Our data suggest a molecular mechanism to explain the coordinated functions played by ISWI and PARP in the regulation of chromatin organization in vivo, providing the first example, to our knowledge, of post-translational regulation of an ATP-dependent remodeler function by poly-ADP-ribosylation.

## Results

### ISWI Localizes to Distinct Domains from PARP and PARylated Chromatin Proteins on Polytene Chromosomes

*Drosophila* has a single PARP gene that spans more than 150 kb of transposon-rich centromeric heterochromatin, which is highly related to the mammalian PARP-1 gene [15]. The NAD^+^-dependent activity of PARP is reversed by PARG, a poly-ADP-ribose glycohydrolase [15]. PARP activity in flies has been associated with the loosening of chromatin structure that precedes gene expression at heat shock puffs [19]. On the contrary, ISWI preferentially associates with transcriptionally silent chromatin [5,6,13,20,21], indicating that PARP and ISWI may play opposite roles in regulating chromosome structure and gene expression.

**Figure 1:**
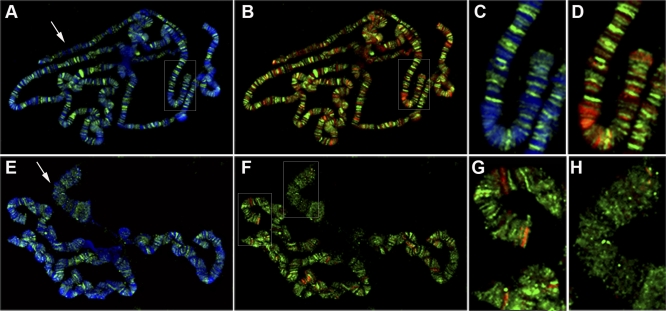
ISWI and PARP Localization on Polytene Chromosomes Is Mutually Exclusive Distribution of PARP and DAPI (A, C, and E) or PARP and ISWI (B, D, F, G, and H) on polytene chromosomes from wild-type (A, B, C, and D) and ISWI^1^/ISWI^2^ (E, F, G, and H) male third instar larvae. PARP mainly localizes in the euchromatic interbands [30], while ISWI pattern contrasts with that of PARP and predominantly overlaps with DAPI stained bands, although a significant fraction of the protein is also associated with interbands [14]. The different colours indicate DAPI (blue), ISWI (red), and PARP (green). (C and D) are magnifications of the boxed areas shown in (A and B), respectively. (G and H) are, respectively, magnifications of the autosome and X-chromosomal areas shown in (F). Arrows in (A and E) indicate X chromosomes.

Given that ISWI and PARP are both chromatin components with enzymatic activity, one possible molecular explanation for the observed genetic interaction (Figure S1) [14] could be that PARP regulates ISWI function by covalently modifying chromatin components to which ISWI binds. Double immunostaining on wild-type polytene chromosomes with antibodies directed against ISWI and PARP shows that localization of the two proteins is largely nonoverlapping (Figure 1B and 1D). We also examined PARP binding in salivary gland polytene chromosomes prepared from transheterozygous *ISWI^1^/ISWI^2^* null mutant male third-instar larvae. In *ISWI* mutant chromosomes, showing chromosome condensation defects (Figure 1F) [5,6], PARP binding levels do not change significantly as compared to wild-type chromosomes (compare Figure 1A and 1B with Figure 1E and 1F).

**Figure 2:**
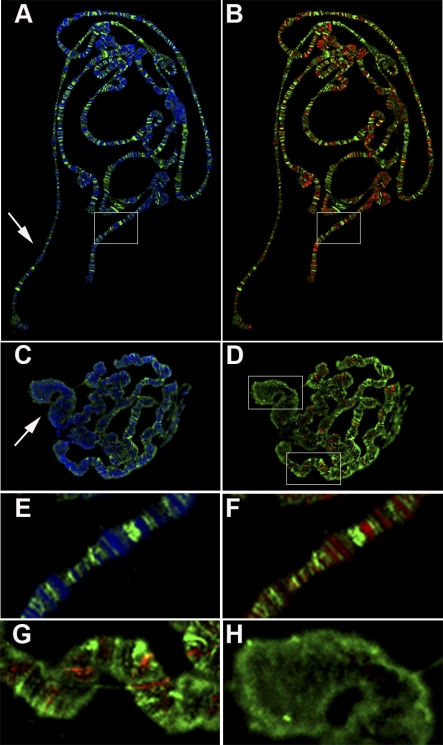
ISWI and PAR Localization on Polytene Chromosomes Is Nonoverlapping Distribution of PAR and DAPI (A, C, and E) or PAR and ISWI (B, D, F, G, and H) on polytene chromosomes from wild-type (A, B, E, and F) and *ISWI^1^/ISWI^2^* (C, D, G, and H) male third instar larvae. The different colours indicate DAPI (blue), ISWI (red), and PAR (green). (E and F) are magnifications of the boxed areas shown in (A and B), respectively. (G and H) are, respectively, magnifications of the autosome and X-chromosomal areas shown in (D). Arrows in (A and C) indicate X chromosomes.

The observation that PARP- and ISWI-bound chromatin domains appear to be nonoverlapping does not exclude the possibility that ISWI binds poly-ADP-ribosylated (PARylated) chromatin domains from which PARP has dissociated. Therefore we conducted double immunostaining with antibodies directed against ISWI and poly-ADP-ribose (PAR) to examine how the pattern of ISWI binding to chromatin domains is correlated with that of PARylated chromatin proteins. We found that like PARP itself, PARylated chromatin proteins show mainly nonoverlapping binding patterns with ISWI on wild-type polytene chromosomes (Figure 2B and 2F). As with PARP binding, PARylated protein binding levels in ISWI mutant chromosomes do not change significantly when compared to wild-type chromosomes (compare Figure 2A and 2B with Figure 2C and 2D; see also Figure S2A). Thus, global chromosome distribution of both PARP and its enzymatic product PAR appears inversely correlated with the distribution of ISWI.

### ISWI Misexpression Causes Over-PARylation of Distinct Chromatin Components

**Figure 3:**
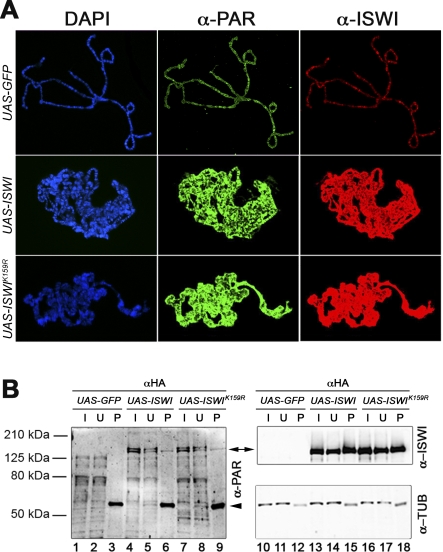
ISWI Misexpression Is Accompanied by Global Hyper-PARylation of Chromosomes (A) Distribution of PAR (green) and ISWI (red) on polytene chromosomes from lines expressing *UAS-GFP*, *UAS-ISWI*, or *UAS-ISWI^K159R^* transgenes under control of the *eyGAL4* driver. Chromosomal DNA was visualized with DAPI (blue). All images were captured with the camera settings used for the UAS-GFP control. To measure directly the ISWI and PAR staining on polytene chromosomes, the levels of ISWI and PAR have been normalized with anti-Mod [14]. Our analysis revealed that on average there is an ∼8.6- and a ∼16.8-fold increase of PAR and ISWI staining, respectively, on polytene chromosomes overexpressing *UAS-ISWI* or *UAS-ISWI^K159R^*, when compared to UAS-GFP chromosomes. (B) Immunoprecipitation with anti-HA antibodies, of salivary gland extracts derived from lines expressing GFP, HA-tagged-ISWI, or -ISWI^K159R^ transgenes under control of *eyGAL4*. Western blot analysis was performed on the input (I: 3% of total), unbound (U: 3%), and pellet (P: 33%) fractions, with aPAR (lanes 1–9), aISWI (lanes 10–18), and aAlpha tubulin (as loading control) antibodies. Arrowhead indicates IgG.

To better understand the functional relationship existing between the nuclear enzymatic activities of ISWI and PARP, we examined whether PARylation levels and distributions are altered by increasing the levels of chromatin-bound ISWI. Double-immunostaining with antibodies directed against PAR and ISWI shows that ISWI is overloaded on polytene chromosomes misexpressing wild-type ISWI, as compared to control chromosomes misexpressing green fluorescent protein (GFP) (Figure 3A). The increase in chromatin-bound ISWI is accompanied by a massive increase in PARylation of chromosomes. This hyper-PARylation is not dependent on ISWI ATPase activity, because misexpression of the enzymatically inactive ISWI^K159R^ protein has a similar effect (Figure 3A). Thus, over-PARylation of chromosomes probably occurs as a consequence of the physical binding of a nonphysiological amount of ISWI on chromatin rather than as an indirect response to an increase in ISWI activity. One interesting possibility is that increased PARylation of chromatin-bound proteins, which might include ISWI itself, could be a homeostatic response designed to counteract excessive ISWI chromosome binding.

Therefore, we conducted immunoprecipitations on protein extracts derived from salivary glands misexpressing ISWI. Comparison of the inputs of native protein extracts derived from salivary glands misexpressing GFP, and HA-tagged wild-type ISWI or ISWI^K159R^ (Figure 3B, lanes 1,4, and 7), shows that the overall level and pattern of protein PARylation are broadly similar in each. However, two bands, which run above the 125-kDa marker, are particularly abundant in the extracts from glands misexpressing either wild-type or ISWI^K159R^ and are absent in the control expressing GFP (Figure 3B, double arrow). Immunoprecipitation of these extracts with aHA antibody detects a PARylated protein that co-migrates with one of the bands specific to the wild-type and ISWI^K159R^ inputs (Figure 3B, compare lanes 4 and 7 with lanes 6 and 9). Remarkably, this protein migrates at the same molecular weight as the HA-tagged versions of ISWI, as revealed by blotting the same filter with aISWI antibody (Figure 3B, double arrow, compare lanes 6 and 9 with lanes 15 and 18). Our immunofluorescence and immunoprecipitation data indicate that the misexpression of ISWI causes PARylation of specific chromatin components and suggest that a main target of PARylation in ISWI misexpressing extracts could be ISWI itself.

### ISWI Is a Target of Poly-ADP-Ribosylation In Vivo

**Figure 4:**
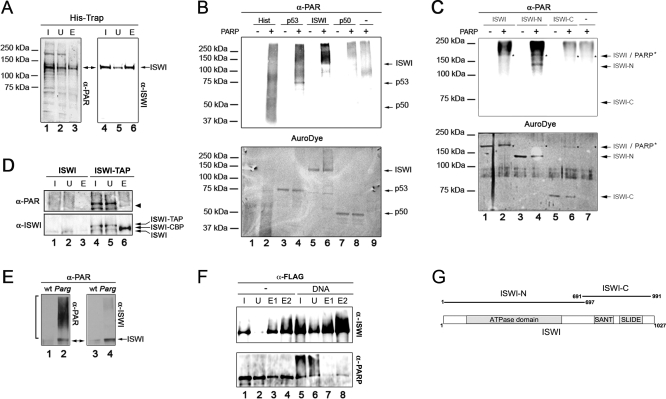
PARylation of ISWI Occurs In Vivo and In Vitro and Is Enhanced in *Parg* Mutants (A) Larval nuclear extracts derived from the HA-6His-tagged ISWI line [6] were affinity purified on a His-Trap column [14] and 3% of the total input extract (I), supernatant (U), and 30% of the eluted fractions were subjected to Western blot analysis with aPAR (lanes 1–3) and aISWI (lanes 4–6) antibodies. The band pointed by the double arrow is lost in a mock purification. (B) Upper panel: aPAR Western blot of purifed histones (lanes 1 and 2), recombinant p53 (lanes 3 and 4), ISWI (lanes 5 and 6), and NFκB p50 subunit (lanes 7 and 8) after incubation in presence (lanes 2, 4, 6, and 8) or absence (lanes 1, 3, 5, and 7) of purified PARP. PARP was also incubated in the absence of any other potential substrate (lane 9). Lower panel: The same filter was stained with AuroDye (GE Healthcare) to reveal the blotted PARylated and non-PARylated proteins. The expected migrations of unmodified p50, p53, and ISWI are indicated by arrows. (C) Upper panel: aPAR Western blot of 2 nmol each of full length ISWI (lanes 1 and 2), N-terminal truncated portion of ISWI (lanes 3 and 4; ISWI-N), C-terminal truncated portion of ISWI (lanes 5 and 6; ISWI-C) after incubation (15 min) in presence (lanes 2, 4, 6) or absence (lanes 1, 3, 5) of purified PARP (0.04 nmol). PARP was also incubated alone in the absence of substrate (lane 7). Lower panel: The same filter was stained with AuroDye (GE Healthcare) to reveal the blotted PARylated and non-PARylated proteins. The expected migrations of ISWI, ISWI-N, and ISWI-C are indicated by arrows, whereas PARP is indicated by asterisks. (D) Larval nuclear extracts derived from larvae expressing TAP-tagged ISWI (ISWI-TAP) and control untagged extracts (ISWI) were affinity purified, as previously described [14]. The ISWI-CBP fusion, consisting of the ISWI protein fused in frame with the calmodulin binding peptide, was eluted from the resin by cleavage with the TEV protease. About 0.05% of the Input extract (I) and supernatant (U), 3% of the eluate (E) were subjected to Western blot analysis with aPAR and aISWI antibodies. Arrows indicate TAP-tagged ISWI (ISWI-TAP), ISWI fused in frame with the calmodulin binding peptide (ISWI-CBP), and endogenous untagged ISWI (ISWI). (E) Immunoprecipitation with aPAR antibodies, of salivary gland extracts derived from wild type (lanes 1 and 3) and *Parg^27.1^* mutant (lanes 2 and 4) lines. Western blot analysis was performed on the immunoprecipitate with anti-PAR (lanes 1 and 2), anti-ISWI (lanes 3 and 4). (F) Pull-down of FLAG-ISWI and purified PARP in the presence (lanes 5–8) or absence (lanes 1–4) of activating DNA. Proteins were detected by Western blot using aISWI and aPARP antibodies. (G) Schematic representation of the domain structure of full length ISWI. The boundaries of the N-terminal (ISWI-N) and C- terminal (ISWI-C) truncated forms of ISWI are depicted.

Since our data indicate that a fraction of overexpressed ISWI can be target of PARylation in vivo, we next investigated whether the same might be true when physiological levels of ISWI are present in the cell. Nickel chelate affinity chromatography conducted on larval nuclear extracts that were derived from a line expressing HA-tagged ISWI fused to 6-histidines at the C-terminal end of the ISWI coding region showed that a fraction of the eluted proteins contains a PARylated band, which migrates at the same size as ISWI (Figure 4A, double arrow on lanes 3 and 6). Consistently, tandem affinity purification (TAP) from larval nuclear extracts expressing a TAP-epitope tagged ISWI [14] reveals a co-eluting PARylated band migrating at the same molecular weight as ISWI (Figure 4D, lane 6, arrowhead). Our data suggest that ISWI could be a physiological target for PARylation in vivo.

If a fraction of ISWI is PARylated in vivo, we might expect this level to increase when the activity of PARP is not counterbalanced by the action of PARG, the poly-ADP-ribose glycohydrolase that reverses the enzymatic activity of PARP [17,18]. We therefore compared the level of ISWI PARylation in wild type and *Parg* mutants. After immunoprecipitation of either wild type or *Parg* mutant salivary gland extracts with antibodies directed against PAR, sequential Western blot with aPAR and aISWI antibodies reveals a PARylated protein migrating at the same molecular weight as ISWI (Figure 4E, lanes 1 and 3). However, both the amount of PARylation and of protein detected by the aISWI antibody are higher in the *Parg* mutant extracts than in those of wild-type origin (Figure 4E, bracket and double arrow on lanes 2 and 4). The immunoprecipitation data we present show that a fraction of ISWI is PARyated under physiological conditions and that ISWI PARylation is increased in *Parg* mutants.

### ISWI Is Poly-ADP-Ribosylated In Vitro

We next explored whether the ISWI PARylation observed in vivo could be recapitulated in vitro. Classic in vitro PARylation assays show that purified PARP is enzymatically active, showing specificity for proteins that are known to be bona fide in vivo and in vitro substrates. Since the number of PAR moieties added to a substrate by PARP is variable and generates branched polymers of ADP-ribose, PARylation in vitro is detected as a signal smearing upward from the molecular weight of the modified target protein. As evidenced by the characteristic smears in an aPAR Western blot of proteins incubated with or without PARP, and the AuroDye staining of the blotted membrane showing the protein migration in the blot, the purified PARP enzyme is able to PARylate histones (Figure 4B, compare lanes 1 and 2) [23] and the recombinant p53 tumor suppressor (Figure 4B, compare lanes 3 and 4) [24] However, PARP shows no activity toward the recombinant p50 subunit of the NFκB complex which is not a target of PARP (Figure 4B, compare lanes 7 and 8) [25], while a low-background signal, derived from self-PARylation, is apparent when the same amount of PARP was incubated alone (Figure 4B, lane 9). Remarkably, when recombinant ISWI and PARP are incubated together, ISWI PARylation is visible as a strong smear that extends upward from the position of unmodified recombinant ISWI (Figure 4B, compare lanes 5 and 6). Thus our PARylation assay clearly indicates that ISWI is a specific substrate of PARP in vitro.

To identify the ISWI protein domain that is the target of PARylation, we set up PARylation assays on recombinant truncated forms of ISWI. *Drosophila* ISWI can be divided in two parts: the N-terminal portion containing the ATPase domain and the C-terminal portion containing the SANT and SLIDE domains (Figure S2B and Figure 4G) [41]. We found that the N-terminal portion of ISWI is specifically PARylated in vitro, whereas the C-terminal portion is not (Figure 4C). Since, both the N- and C-terminal of ISWI contribute to the interaction with nucleosomal DNA [41], our data suggest that PARylation of the N-terminal fragment could target the nucleosomal DNA recognition element of ISWI present in the ATPase domain.

### PARP Associates with ISWI In Vitro

If one of the enzymatic targets of PARP is ISWI, we should be able to monitor a physical interaction between the two proteins in vitro or in vivo. Immunoprecipitations conducted on wild-type larval nuclear extracts using antibodies directed against ISWI, or on extract derived from HA-epitope tagged ISWI using the aHA antibody, failed to detect a direct physical association between ISWI and PARP (unpublished data). Since PARP has been shown to be dynamically associated with its target sites on chromatin [26], we may not be able to detect a direct physical interaction with ISWI from crude nuclear extracts. Therefore, we decided to monitor in vitro the association of PARP with ISWI, by pull-down experiments using purified PARP and FLAG-epitope tagged recombinant ISWI. In the absence of DNA, the aFLAG-coupled resin can pull down FLAG-tagged ISWI together with a discrete amount of PARP (Figure 4F, lanes 3 and 4). When DNA is included in the binding reaction, the enzymatic activity of PARP is stimulated, as show by the self PARylation of PARP and the PARylation of ISWI detected with aPARP and aISWI antibodies, respectively (Figure 4F, compare lanes 1 and 5). Remarkably, in the presence of DNA, the fraction of PARP that is found associated with ISWI is dramatically decreased (Figure 4F, compare lanes 3 and 4 with lanes 7 and 8). Interestingly, the residual fraction of PARP that associates with ISWI in the presence of DNA does not appear strongly PARylated, suggesting that actively PARylating PARP does not preferentially associates with ISWI, probably explaining PARP dynamic association with its chromatin targets observed in vivo as well as our failure to detect PARP–ISWI interaction by immunoprecipitation from crude protein extracts (Figure 4F, compare lanes 5 with 7 and 8).

### PARylation of ISWI Inhibits Its ATPase Activity

**Figure 5:**
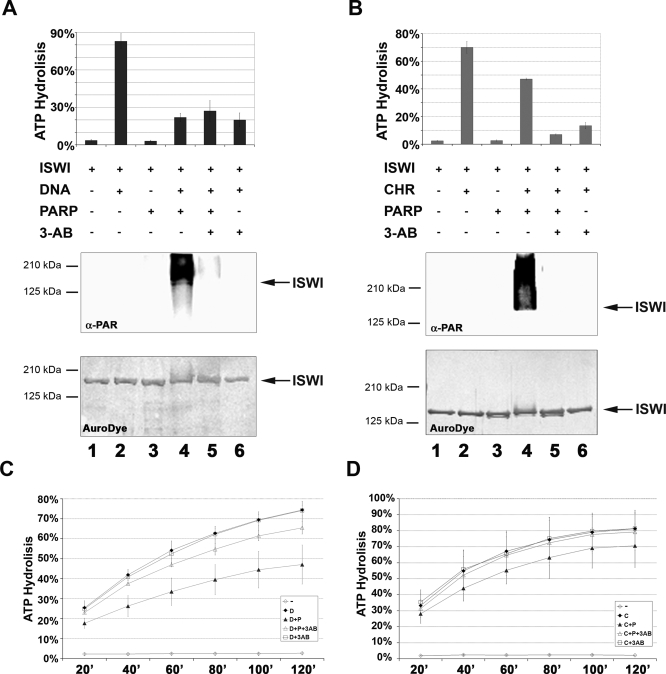
PARylation of ISWI Inhibits Its In Vitro ATPase Activity (A) Dual assay for DNA-dependent ATP hydrolysis and poly-ADP-ribosylation (molar ratio ISWI/PARP ∼2.5). Black bars indicate percentage of total ATP hydrolysed, as measured by TLC analysis of an aliquot from each reaction mixture (1–6). Inclusion (+) or omission (-) of ISWI, DNA, PARP and 3-AB during the reaction is indicated. Aliquots of the same reaction mixtures were analysed by Western blot with the anti-PAR antibody. As a loading control, the same filter was stained with AuroDye. Results of analyses for the individual reaction mixtures are vertically aligned. (B) Dual assay for chromatin-stimulated ATP hydrolysis and poly-ADP-ribosylation (molar ratio ISWI/PARP ∼2.5). Grey bars indicate % of total ATP hydrolysed, as measured by TLC analysis of an aliquot from each reaction mixture (1–6). Inclusion (+) or omission (-) of ISWI, chromatin (CHR), PARP, and 3-AB during the reaction is indicated. Aliquots of the same reaction mixtures were analysed by Western blot with anti-PAR antibody. As a loading control, the same filter was stained with AuroDye. Results of analyses for the individual reaction mixtures are vertically aligned. (C) Time course of DNA-dependent ATP hydrolysis (molar ratio ISWI/PARP ∼133). Open diamonds (-): reactions contained PARP and ISWI, but not DNA. Black diamonds (D): reactions contained ISWI and DNA, but not PARP. Black triangles (D+P): reactions contained ISWI, PARP, and DNA. Open triangles (D+P+3-AB): reactions contained ISWI, PARP, DNA, and 3-AB. Open squares (D + 3-AB): reactions contained ISWI, DNA and 3-AB, but not PARP. (D) Time course of chromatin-stimulated ATP hydrolysis (molar ratio ISWI/PARP ∼133). Open diamonds (-): reactions contained PARP and ISWI, but not chromatin. Black diamonds (C): reactions contained ISWI and chromatin, but not PARP. Black triangles (C+P): reactions contained ISWI, PARP and chromatin. Open triangles (C+P+3-AB): reactions contained ISWI, PARP, chromatin and 3-AB. Open squares (C + 3-AB): reactions contained ISWI, chromatin and 3-AB, but not PARP. The apparent mild ATPase inhibition exerted by PARP, under these conditions, is due to the very high ISWI/PARP molar ratio present in the reaction.

To investigate whether PARylation influences ISWI ability to remodel nucleosomes, we first tested whether ISWI ATPase activity is modified in the presence of PARP activity. As PARP and ISWI enzymatic activities are differentially stimulated by linear DNA or nucleosome arrays [20], we conducted a dual assay to test ATPase and PARylation activities in the presence of recombinant ISWI together with purified PARP (with a molar ratio ISWI/PARP ∼2.5), using both linear DNA and in vitro assembled chromatin as stimulators of these enzymatic activities (Figure 5A and 5B and Figure S3A and S3B). Both the ATPase of ISWI and the PARylation activity of PARP were assayed from the same reaction mixture for each condition tested.

Interestingly, ISWI activity in the presence of PARP is reduced by ∼60% for DNA-dependent (Figure 5A, compare lanes 2 and 4), and ∼40% for nucleosome-stimulated ATPase activities (Figure 5B, compare lanes 2 and 4). The observed inhibition correlates with PARylation of ISWI, as indicated by aPAR Western blot analysis and the AuroDye staining of the blotted membrane of the same samples. We were unable to reverse the PARP-dependent ATPase inhibition of ISWI in the presence of 3-aminobenzammide (3-AB), a competitive inhibitor of PARP, in the reaction. However, this is because the amount of 3-AB that is sufficient to inhibit PARP activity (Figure 5A and 5B, lane 5), also strongly inhibits ISWI ATPase activity (Figure 5A and 5B, lane 6).

Therefore, to verify that the ISWI ATPase inhibition was caused specifically by PARP activity, we lowered ∼50× the amount of PARP in the ATPase/PARylation assay (molar ratio ISWI/PARP ∼133). At these levels, PARP can be inhibited by 3-AB concentrations that are not inhibitory for ISWI ATPase activity (Figure 5C and 5D, open square graph). Even this very low amount of PARP exerts an inhibitory effect on both ISWI DNA-dependent and chromatin-stimulated ATPase activity over a 120-min time course (Figure 5C and 5D, compare filled diamond with filled triangle graphs). Furthermore, under these conditions, 3-AB reverses the effect of PARP, indicating that ISWI ATPase inhibition is specifically caused by PARP activity (Figure 5C and 5D, open triangle graph).

Although chromatin stimulates the ATPase activity of ISWI ∼10 fold more then DNA, we observed a lower inhibition of ISWI by PARP in the presence of chromatin. However, a lower inhibition with the substrate that is able to stimulate more the ATPase activity of ISWI is expected. In fact, during the ATPase/PARylation reaction, the fraction of ISWI that is not yet PARylated should have a higher ATPase activity in the presence of chromatin than with DNA, thus explaining the lower inhibition observed in the presence of chromatin in our assay.

During the ATPase/PARylation reaction, PARP can also generate free soluble PAR [17,18]. The PAR that is not covalently attached to ISWI could in theory account for the observed PARP-dependent ISWI ATPase inhibition. We show that under the conditions in which we conducted the ATPase/PARylation assay, free PAR cannot account for the observed ISWI ATPase inhibition (Figure S4). Thus, we conclude that it is indeed the specific PARylation of ISWI that inhibits its DNA and nucleosome-stimulated ATPase activity.

### PARylation of ISWI Inhibits DNA and Nucleosome Binding

The inhibition of ISWI ATPase activity by PARP could indicate either that the PARylation of ISWI directly blocks its ATPase activity after DNA or nucleosome recognition, or that this posttranslational modification counteracts ISWI ability to productively interact with its substrates. To distinguish between such alternatives and gain deeper insight into the molecular mechanism underlying the inhibition of ISWI ATPase upon PARylation, we conducted nucleosome shift assays in which a mononucleosome-enriched polynucleosome fraction purified by sucrose gradient was used as binding substrate with increasing amounts of ISWI, in the presence or absence of PARP (Figure S5A).

**Figure 6:**
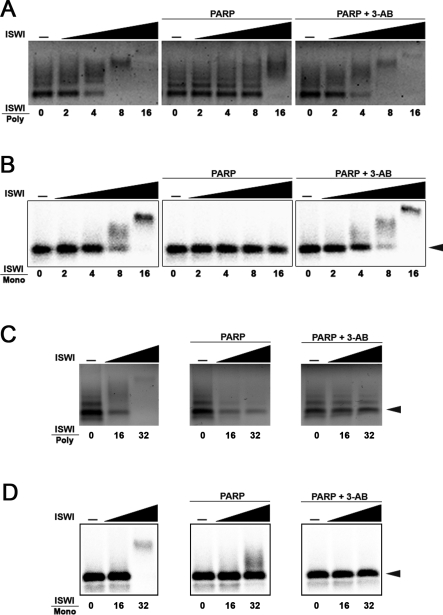
PARylation of ISWI Inhibits Nucleosome Binding In Vitro Gel retardation assays of (A) mixed-length poly-nucleosomes purfied from chicken erythrocyte or in vitro assembled monoucleosomes (B) after incubation with increasing amounts of ISWI, in the presence or absence of PARP and in the presence or absence of PARP plus its competitive inhibitor 3-AB. For purified nucleosomes, 125 ng of a mixed length poly-nucleosome fraction were incubated with 2, 4, 8, and 16 nmol of ISWI, 0.4 nmol of PARP and 5 mM 3-AB. For reconstituted recombinant nucleosomes, 0.25 nmol of mononucleosomes were incubated with 0.5, 1, 2, and 4 nmol of ISWI, 0.2 nmol of PARP, and 2.5 mM 3-AB. Activating DNA together with increasing amounts of ISWI were tested for the ability to shift (C) purified polynucleosomes and (D) recombinant mononucleosomes before and after incubation with PARP or PARP and 3-AB. Sixteen, and 32 nmol of ISWI, 0.4 nmol of PARP, and 40 mM 3-AB were used for purified nucleosomes, while 4 and 8 nmol of ISWI, 0.2 nmol of PARP, and 20 mM 3-AB were used for reconstituted recombinant nucleosomes. Numbers indicate mass ratio of ISWI over purified polynucleosomes (ISWI/Poly) or over in vitro assembled mononucleosomes (ISWI/Mono).

Under the reaction conditions used, in the absence of PARP, a molar excess of ∼8 fold ISWI/nucleosomes is sufficient to shift the migration of the bulk of the nucleosome population (Figure 6A). In contrast, when PARP is included in the bandshift reaction, the mass excess of ISWI necessary to shift all the nucleosomes is at least 2-fold greater (Figure 6A). These data indicate that the PARylation of ISWI inhibits its ability to interact with arrays of nucleosomes.

To exclude the idea that posttranslational modifications of the histones—present in the purified polynucleosome fraction used as substrate—contribute to this effect, we repeated the bandshift experiments using a mononucleosome that was assembled in vitro by salt dialysis (Figure 6B). As with the polynucleosomes, a molar excess of less than 8-fold ISWI/nucleosome is sufficient to up-shift the recombinant mononucleosome if PARP is absent (Figure 6B). However, when PARP is added to the bandshift reaction, there is no sign of change in migration of the DNA–protein complexes even at ISWI/nucleosome mass excess of ∼16-fold and a molar ratio ISWI/nucleosome of ∼32-fold is necessary to start to appreciate a nucleosome shift (Figure 6B and unpublished data). Both the inhibition of PARP on ISWI binding to the purified poly-nucleosomes, and the more pronounced effect on recombinant mononucleosomes, are completely reversed by the addition of 3-AB (Figure 6A and 6B).

Core histones present in purified polynucleosomes, and in vitro assembled mononucleosomes can be themselves a target of PARylation and contribute to the reduction in nucleosome affinity observed with ISWI and PARP. However, ISWI DNA bandshift assays in the presence of PARP show a reduction in DNA affinity similar to the one observed with nucleosomal DNA (Figure S3D), indirectly suggesting that histone PARylation does not contribute to the reduction in nucleosome affinity observed with PARylated ISWI.

To exclude directly the idea that histone PARylation might contribute to the observed loss of affinity of ISWI for nucleosomes in the presence of PARP, we first PARylated ISWI and then conducted the nucleosome binding step in the presence of 3-AB, to prevent PARylation of histones (Figure S5B). The presence of DNA, to first stimulate PARP activity in the absence of nucleosomes, causes an apparent loss of affinity of ISWI for purified polynucleosomes or in vitro–assembled mononucleosomes due to binding competition. Indeed, a 32-fold ISWI/nucleosome is necessary to shift poly- and mononucleosomes (Figure 6C and 6D). As previously observed, when PARP is added to the bandshift reaction under conditions in which both histones and ISWI could be target of PARylation, there is no sign of poly- and mononucleosome shift even with a 32-fold ISWI/nucleosome ratio (Figure 6C and 6D). Remarkably, when ISWI is PARylated and 3-AB is included to prevent PARylation of histones, we observe a loss of affinity of ISWI with poly- and mononucleosomes that is indistinguishable from conditions when both histones and ISWI could be subjected to PARylation (Figure 6C and 6D). Our data strongly suggest that PARylation of ISWI is sufficient to reduce its affinity with nucleosomal DNA, which in turn may be responsible for the observed reduction in ATPase activity.

### PARP Activity Counteracts ISWI Function In Vivo

**Figure 7:**
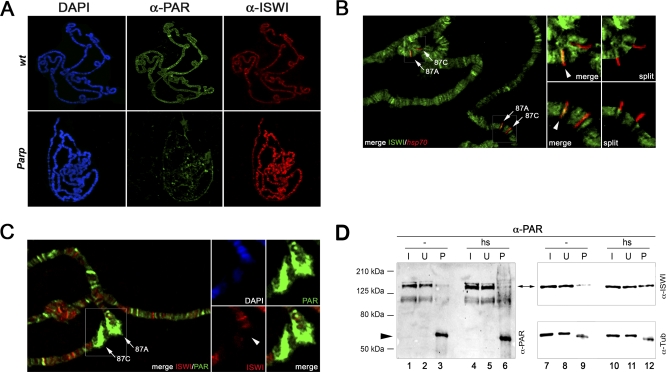
PARP Activity Counteracts ISWI Function In Vivo (A) Distribution of PAR (green) and ISWI (red) on polytene chromosomes from wild-type (wt) and homozygous *Parp^C03256^* (*Parp*) male third instar larvae. Chromosomes were also stained with DAPI to visualize DNA (blue). To measure directly the ISWI and PAR staining on polytene chromosomes, the levels of ISWI and PAR have been normalized with anti-Mod [14]. Our analysis revealed that on average there is a ∼4.1-fold decrease of the PAR staining in the *Parp^C03256^* mutant that is accompanied by a ∼6.6-fold increase in ISWI staining, when compared to wt chromosomes. (B) ImmunoFISH using an aISWI antibody (green) and a DNA probe for the *hsp70* loci at 87A and 87C (red) was carried out on polytene chromosomes prepared from wild-type third instar larvae maintained under non–heat-shocked conditions. Large panel shows the merged image for both signals. The image contains two examples of cytological loci 87A and 87C, which are indicated by arrows (large panel). Anti-ISWI immunostaining and in situ hybridization with fluorescently labeled probe are shown for magnifications of the regions harboring these loci (boxed areas), as both merged and split chromosome images (small panels). Arrowheads indicate the binding of ISWI at the 87A *hsp70* locus. (C) Co-immunolocalization of ISWI and PAR with DAPI staining on polytene chromosomes prepared from wild-type third instar larvae exposed to heat shock. Large panel shows the merged signals for ISWI (red) and PAR (green). A pair of heat shock puffs at 87A and 87C are indicated by arrows (large panel). Magnifications of the region containing both puffs are shown for DAPI, PAR, ISWI, and the ISWI/PAR merge (small panels). Arrowhead indicates loss of ISWI binding at the 87A *hsp70* locus. (D) Immunoprecipitation with aPAR antibody on wild-type native salivary gland extracts prepared under non–heat-shock (lanes 1, 2, 3, 7, 8, and 9) or heat-shock conditions (lanes 4, 5, 6, 10, 11, and 12). Western blot analysis was performed on the input (I: 3% of total), unbound (U: 3%), and pellet (P: 50%) fractions with anti-PAR (lanes 1–6), anti-ISWI (lanes 7–12), and anti-Tub (as loading control) antibodies. Arrowhead indicates IgG.

If PARP inhibits ISWI ATPase activity, by reducing nucleosome affinity through ISWI PARylation as suggested by our in vitro data, we would predict that the loss of PARP function in vivo might result in an increase of chromatin-bound ISWI. We carried out double immunostaining with antibodies directed against PAR and ISWI and compared the chromatin binding patterns obtained from *Parp* mutants with those of wild-type polytene chromosomes. Remarkably, the levels of ISWI are significantly higher in *Parp* mutant chromosomes than in wild type (Figure 7A). Thus, loss of PARP activity appears to cause a global increase of chromatin bound ISWI on polytene chromosomes.

Given that PARylation of ISWI causes a reduced affinity for nucleosomes in vitro, and that loss of PARP function is correlated with an increase in ISWI chromatin binding, we might also expect that an increase in PARP activity at specific chromosome domains should result in loss of ISWI binding from the same domains. The dual detection by immuno-FISH of the *hsp70* genes at cytological location 87A and 87C and ISWI has allowed us to establish that in the absence of heat shock conditions, ISWI very reproducibly binds the *hsp70* locus at 87A (Figure 7B, arrowhead). However, upon heat shock, the *hsp70* loci acquire elevated levels of PARylated chromatin components and PARP is required in the process of chromatin decondensation that manifests itself as localized heat shocked puffs (see DAPI in Figure 7C and Figure S6A and S6B) [19]. Remarkably, after PARylation of chromatin is induced by heat shock, among other loci we observe that the specific binding of ISWI at 87A is lost (Figure 7C, arrowhead). If, upon heat-shock, PARP activity removes ISWI chromatin binding at the 87A *hsp70* locus, we would predict that in the *Parp* mutant, ISWI should remain bound to chromatin after heat shock. Indeed, in *Parp* mutant polytene chromosomes ISWI remains bound to the *hsp70* locus 87A after heat shock (Figure S6C and S6D). In line with these findings, in heat-shocked salivary gland protein extracts, the amount of PARylated ISWI is significantly higher than in extracts obtained under non–heat-shocked conditions (Figure 7D).

Our data indicate that binding of ISWI to the *hsp70* gene can be counteracted by PARylation of chromatin at the 87A locus and that the loss of ISWI binding upon heat shock is a physiological response that is directly dependent upon the activity of PARP. Since ISWI is a target of PARylation both in vitro and in vivo, we propose that upon heat shock, ISWI bound at the *hsp70* locus could be among the chromatin components that get PARylated and this in turn promotes its release from chromatin.

## Discussion

### PARP and ISWI Could Compete for Common Chromatin Target Sites

PARP is an abundant nuclear protein that plays important roles in multiple DNA repair pathways [17]. Interestingly, high PARP enzymatic activity has also been observed in chromosomal sites where high transcriptional activity is occurring [17]. One main goal in the study of the diverse physiological roles of PARP is the identification of molecular determinants that can stimulate PARP, besides DNA damage. Recently, it has been shown that PARP activity, in the absence of DNA damage, can be stimulated by transcription-coupled, TopoIIb-dependent transient double-stranded DNA breaks [27].

Interestingly, nucleosomal histone H4 amino terminal tails have been shown to also activate PARP activity in vitro [26]. Remarkably, the amino termini of histone H4 also stimulates ISWI activity [13,28], whereas PARP's NAD^+^-dependent activity is known to be inhibited by ATP, a substrate of ISWI [29]. Moreover, ISWI and PARP have opposing effects on the binding of the linker histone H1 to chromatin; human PARP-1 competes with H1 for binding to the nucleosome linkers [30,31], while ISWI promotes chromosome condensation through the loading of histone H1 [5]. PARP and H1 exhibit a reciprocal pattern of chromatin binding at many RNA polymerase II–transcribed promoters [31]. Therefore, one intriguing possibility is that PARP activity can counteract ISWI function. Indeed, ISWI and PARP appear to compete for common chromatin target sites, as supported by their nonoverlapping chromatin binding patterns. The antagonistic dominant action of PARP over ISWI may help to promote transcription at specific chromatin sites by opening chromatin and blocking higher order chromatin structure formation by ISWI, although PARP has also been shown to directly promote the formation of compact, transcriptionally repressed chromatin [30].

### Poly-ADP-Ribosylation Can Regulate ATP-Dependent Nucleosome Remodeler

We found that ISWI is PARylated in vivo and in vitro, revealing the molecular basis of the genetic interaction we found between *ISWI* and *Parp* [14]. We show that PARylation of ISWI inhibits both its ATPase activity and its chromatin binding, in vitro and in vivo. Although, the PARylation of ISWI reduces its chromatin-stimulated ATPase activity by ∼40%, PARylated ISWI reduces its affinity for nucleosomes by nearly 10-fold. These differences can be explained by the different nature of the ATPase/PARylation and band shift assays. In contrast with the band shift assay, during the enzymatic ATPase/PARylation assay, the fraction of ISWI that is not yet PARylated can be strongly and rapidly stimulated by nucleosomes, thus masking the strong inhibition exerted by PARP on the PARylated ISWI fraction.

PARP is in a dynamic equilibrium between its chromatin-bound and free nucleoplasm form [26]. Indeed, our in vitro data indicate that the interaction between active PARP and ISWI is likely to be transient, highlighting the dynamic nature of this functional interaction. PARP can promote or inhibit chromatin binding of a variety of nuclear factors [32,33]. The finding that PARylation of ISWI lowers its affinity for chromatin suggests a molecular explanation for the mutually exclusive patterns of ISWI and PARP on wild-type polytene chromosomes, although other mechanisms probably regulate chromatin binding of the two proteins in vivo.

Cross-linking studies have shown that ISWI contacts the nucleosome at two locations: (i) on the linker DNA near the nucleosome entry/exit site, and (ii) at an internal site about two turns from the nucleosomal dyad [34]. Because ISWI needs some overhanging piece of DNA to remodel nucleosome, the inhibition of both ISWI-specific DNA and nucleosome stimulated ATPase upon PARylation probably reflects the reduced affinity of PARylated ISWI for free DNA and nucleosomal DNA that we observed in vitro. Indeed, under the conditions we conducted the *ISWI^K159R^* screen, we found that the EP3570 insertion in the *Parp* gene can increase the level of chromatin PARylation, thus reducing ISWI binding on polytene chromosome. On the other hand, the observation that over-expression of ISWI leads to an increase in chromatin-bound PARylated ISWI does not contradict this model; PARylation of ISWI only lowers its affinity for chromatin, but does not entirely prevent it from binding in vitro.

It is generally accepted that PARP plays roles in both local chromatin remodeling and the recruitment/modulation of the activity of various factors involved in DNA replication, repair, transcription, and recombination [17,18].

Our work presents the first example of a nucleosome remodeling activity being regulated by PARylation and the first insight into the mechanism of regulation of a chromatin remodeler by PARP activity. Although our data suggest that the modulation of ISWI activity by poly-ADP-ribosylation could be a key regulatory step acting in the context of the heat-shock induction of the *hsp* loci in flies, further studies will be necessary to fully understand the evolutionary conservation of this mechanism and other physiological context in which ISWI regulation by PARP occurs.

## Materials and Methods

### *Drosophila* stocks and genetic crosses.

Flies were raised at 25 °C on K12 medium [35]. Unless otherwise stated, strains were obtained from Bloomington Stock Center and are described in FlyBase (http://www.flybase.org). For immunofluorescence staining and protein extract preparations, ISWI^1^/ISWI^2^ male larvae were obtained as previously described [14]. Salivary glands misexpressing GFP, wild-type ISWI, and mutant ISWI^K159R^ were obtained as previously described [5]. *Parp^C03256^* is a new loss-of-function allele of *Parp* that survive until the third instar larval stage. The *Parg^27.1^* allele was generated as described [36].

### Immunostaining of polytene chromosomes and immuno-FISH.

For immunostaining of polytene chromosomes with antibody against monoclonal aPAR, slides were washed for 1 min in cold 96% EtOH; then the squashed areas were covered with 10% cold TCA for 10 min. Slides were subsequently washed for 1 min in 70% EtOH, 1 min in 90% EtOH, 1 min in 96% EtOH, and blocked. For immunostaining of polytene chromosomes with antibodies against ISWI and PAR, the slides were blocked in PBS, 3 % BSA, 0.1 % Triton X-100, whereas for PARP staining and ISWI/PARP double staining, slides were blocked in PBS, 5% nonfat milk, and processed as described previously [21,30]. Immuno-FISH was conducted after immunostaining with ISWI and PAR antibodies, as described previously [37], except that denaturation was done for 8 min; the hsp-70 probe was labeled using Biotin-Nick translation Mix (Roche).

### Protein extracts and Western blot procedures.

Salivary gland protein extracts misexpressing GFP, wild-type ISWI, ISWI^K159R^, and from wild-type or ISWI^1^/ISWI^2^ mutants were obtained as previously described [14]. Larval nuclear extracts were prepared as described [38]. After SDS-PAGE, proteins were transferred to nitrocellulose membrane (Whatman Schleicher & Schuell) and stained with AuroDye Forte (GE Healthcare). Proteins were detected by Western blot using SuperSignal West Femto substrate (Pierce). Chemiluminescent signals were acquired with the ChemiDoc XRS imager (BioRad).

### Immunoprecipitation, affinity chromatography, and pull-down experiments.

1.5 μg of anti-HA (Roche) or anti-PAR (10H) antibodies were pre-bound to 30 μl of Protein A-Sepharose 4B Fast Flow resin (Sigma). The resin was incubated with 250 μg of salivary gland protein extracts from wild-type third instar larvae under non–heat shock or heat shock conditions, or third instar larvae misexpressing GFP, wild-type ISWI, or *ISWI^K159R^*, or with total extracts derived from 30 wild-type or 30 *Parg^27.1^/Y* mutant adult males. The affinity purification of His-tagged and TAP-tagged ISWI were conducted as described in [14]. In pull-down experiments, 8 nmol of FLAG-ISWI [20] were incubated with 8 nmol of purified PARP-1 (Trevigen) in 1X PARP cocktail, 8ml 100mM Tris-HCl pH8, 1mM MgCl_2_, 1 mM DTT with or without 1mg of activated DNA (Trevigen) for 15 min at 25°C. For each condition tested, FLAG-ISWI was pulled-down with 50 ml of “Anti-FLAG M2 Affinity Gel Freezer-Safe” (Sigma).

### Poly-ADP-ribosylation in vitro assay.

Unless otherwise indicated, the standard reaction contained 4 nmol of one of the following proteins: ISWI, p53 (Santa Cruz Biotech), p50 NFκB (Promega), and histones in 1X PARP Buffer, 1 X PARP Cocktail, 1μg activated DNA with or without 1nmol of PARP-1 in a final volume of 12.5 μl (Trevigen). Samples were incubated for 1 h at 25 °C, then reactions were stopped by the addition of 3X SDS loading buffer and analyzed on 8% SDS-PAGE.

### ATPase assay.

The standard reaction (14 μl) contained 4 nmol of ISWI, 6.6 mM HEPES (pH 7.6), 0.66 mM EDTA, 0.66 mM 2-mercaptoethanol, 0.033 % NP-40, 1.1 mM MgCl_2_, 33 μM ATP, 5 μCi [γ-^33^P]ATP-3000 mmol-1 (GE Healthcare). Either 1 μg of activated DNA (Trevigen) or 100 ng of in vitro assembled chromatin [39] was used as substrate. In ATPase assays containing PARP, the mix was added with 1X PARP cocktail and 8 μl 100mM Tris-HCl pH 8, 1mM MgCl_2_, 1mM DTT. The chromatin used as substrate in these assays was treated with UV (290–320 nm) for 20 min on ice. In ATPase time course assays, 0.03 nmol of PARP and 0.14 mM 3-AB were used. In all other ATPase assays, the samples contained 1.5 nmol PARP and 14 mM 3-AB. Unreacted ATP and free γ-phosphate were separated by thin layer chromatography as previously described [20]. ATP hydrolysis quantification was done with the Personal Molecular Imager FX System (BioRad).

### Nucleosome bandshift assay.

Poly-nucleosomes were prepared from chicken erythrocytes by sucrose gradient [40]. Mononucleosomes were assembled by salt gradient dialysis on 146-bp labeled DNA fragments as previously reported [41]. Increasing amount of ISWI were incubated in the presence or absence of PARP, 3-AB, poly-nucleosomes, or in vitro–assembled mononucleosomes in 5 μl of 100mM Tris-HCl pH 8, 1mM MgCl_2_, 1mM DTT, 1X PARP cocktail for 15 min at 25 °C in a final volume of 10 μl. Then 5 μl of 50 mM Tris-HCl (pH 8), 50 mM NaCl, 1 mM MgCl_2_, 100 μg/ml chicken albumin, 0.05 % NP-40, 10 % glycerol, were added to each sample and the reaction was incubated for 10 additional min at 25 °C. To poly-ADP-ribosylate ISWI in absence of nucleosomes, ISWI was first incubated with 50 ng of activated DNA and PARP in 5 μl of 100 mM Tris-HCl pH 8, 1 mM MgCl_2_, 1 mM DTT, 1X PARP cocktail for 15 min at 25 °C. Then 3-AB was added to the mix, together with 125 ng poly-nucleosome or 0.5 nmol of mononucleosomes. Poly-nucleosomes were resolved on 1.4% agarose gel in 0.3 X TBE at 4 °C for 50 min as previously reported [41] and detected by EtBr staining. Gels containing labeled mononucleosomes were dried and detected by Personal Molecular Imager FX System (BioRad).

## Supporting Information

Figure S1 *ISWI* Genetically Interacts with *Parp* and *Parg*
Loss of *ISWI* function, by eye-specific misexpression of the dominant negative allele *ISWI^K159R^*, produces catalytically inactive ISWI that is incorporated into native complexes giving rise to rough and reduced eye phenotypes in otherwise healthy flies [6,13,20,21]. We used this in vivo eye assay to conduct an unbiased genetic screen for mutations in genes that dominantly modify phenotypes resulting from loss of *ISWI* function in the eye [14]*.* The rationale of the genetic screen we conducted is that if a mutation in one gene can dominantly modify the phenotype resulting from misexpression of *ISWI^K159R^* in the eye, it is likely that the protein encoded by the mutated gene is involved in the same biological process as *ISWI.* We screened a collection of ∼2,300 *Drosophila* lines bearing the special EP modified transposable P-elements that can lead to the interruption of a gene or to GAL4-dependent misexpression of the genes adjacent to the insertion site [14].
(A) Among the loci identified in our screen, we recovered a single EP-element insertion–*EP3570*–that maps the natural transposon *1360* present in the first intron of the *Parp* gene. The EP3570 line was “cleaned” for a second insertion on a euchromatic locus on the third chromosome, and was subsequently mapped by FISH and iPCR using standard protocols (unpublished data). The arrowhead marks the *Parp^CH1^* mutation, a previously described *Parp* allele [15], while the directional arrow indicate the *EP3570* insertion.
(B) To validate the observed genetic interaction, we tested if other alleles of *Parp* and *Parg* genetically interacted with *ISWI^K159R^*. Indeed, similarly to the EP insertions recovered in the screen, the loss-of-function alleles *Parp^CH1^* and *Parg^27.1^* both dominantly enhanced *ISWI^K159R^* eye defects. The severity of rough/reduced eye phenotypes was scored on a scale of 1 to 6 (with 6 corresponding to the most severe; [14]). The line *Df(yw)* was used as a wild-type negative control; The Kolmogorov-Smirnov two-sample test (KS-test) was applied to calculate if the cumulative frequency distributions of the eye scores of the experimental and control progeny classes were statistically different (*p* = 0.05). For the KS-test we used the *Df(yw)* eye distributions or the balancer class, when available.
(C) Remarkably, a single insertion near the *Parg* gene–*EP1623–* was also found to act as a weak *ISWI^K159R^* enhancer [14]. *Parg^27.1^* is a previously described *Parg* allele [36]. The genome maps for *Parp* and *Parg* were obtained from FlyBase (http://www.flybase.com).
(D) Complementation tests between *Parp^CH1^*/*TM3, Sb*, and *EP3570*/*TM6B, Hu, Tb* or *Parg^27.1^*/FM7 and EP1623 lines show that while the *EP1623* insertion fully complemented the *Parg^27.1^* loss-of-function allele, the *EP3570/Parp^CH1^* progeny is semi-lethal, suggesting that the *EP3570* insertion generates a partial loss-of-function allele of *Parp*. Numbers indicate scored progeny for each genotype.
(E) Double-immunofluorescence for PAR and ISWI on polytene chromosomes extracted from *eyGAL4/+; EP3570/+* larvae are compared with control polytene chromosomes misexpressing the GFP (*eyGAL4/+; UAS-GFP/+*). The eyG*AL4;EP3570* polytene chromosomes show an increased level of PARylation compared to control chromosomes (∼200%). Furthermore, the increase in PARylation in *eyGAL4;EP3570* polytene chromosome is accompanied by a substantial reduction (∼50%) of ISWI binding, not associated with chromosome condensation defects. The levels of ISWI and PAR in the eyG*AL4;EP3570* and *eyGAL4;UAS-GFP* polytene chromosomes have been normalized with anti-Mod [14] and do not change by Western blot on larval nuclear extracts (unpublished data). Our data suggest that the eyGAL4 misexpression of PARP in the *EP3570* increases general chromatin PARylation that in turns reduces ISWI binding to chromatin, thus causing the enhancement of *ISWI^K159R^* eye phenotypes we observed with the *EP3570* line. These data bring support to the notion that the antagonistic relationship between ISWI and PARP, which is presented in this manuscript, is physiological and of biological significance.
(3.46 MB TIF)

Figure S2 Analysis of PARylated Proteins in Wild-Type and *ISWI* Mutants and of Recombinant ISWI Proteins (A) Aliquots of total protein extracts from wild-type (wt) and the *ISWI^1^/ISWI^2^* (*ISWI*) mutant third instar larvae were separated by SDS-PAGE and subjected to Western blot analysis, with anti-PAR (lanes 1 and 2) and anti-ISWI (lanes 3 and 4) antibodies. Filters were stripped and blotted with anti-alpha-tubulin antibodies to serve as a loading control. It should be noted that two of the most strongly PARylated protein bands in total protein extracts from wt salivary glands are significantly underrepresented in *ISWI* mutant extracts. Asterisks mark the two bands that are underrepresented in *ISWI* salivary glands extracts. The arrowhead indicates a PARylated band that migrates at the same molecular weight as ISWI.
(B) Recombinant wt (lane 1), N-terminal (lane 2), and C-terminal (lane 3) ISWI produced in E. coli as previously described [41].
(367 KB TIF)

Figure S3 Characterization of UV-Treated Chromatin; Effect of ATP on PARP Activity; PARylation of ISWI Reduces ISWI DNA Binding (A) ATP hydrolysis assay of recombinant ISWI incubated with: no DNA or chromatin (1); linear DNA (2); non–UV-treated chromatin (3); or UV-treated chromatin (4).
(B) Anti-PAR Western blot to assay PARylation of recombinant ISWI incubated with: no DNA or chromatin (1): linear DNA (2); non–UV-treated chromatin (3); UV-treated chromatin (4). UV-treated chromatin stimulated PARP activity better than non–UV-treated chromatin, therefore we used the former as chromatin substrate for the ATPase/PARylation in vitro assay.
(C) Upper panel: PARP (0.5 nmol) auto-PARylating activity starts to get inhibited with ATP concentration as low as 2.5 mM, (compare lanes 5 and 6). However, the trans-PARylating activity of PARP over ISWI (1 nmol) is not affected even at ATP concentration of the order of 10 mM (compare lanes 1, 2, 3, and 4). PARylation in vitro assays were allowed to proceed for 15 min. Lower panel: The same filter was stained with AuroDye (GE Healthcare) to reveal the blotted PARylated proteins.
(D) Gel retardation assays with a mixed-length population of DNA fragment with average size of 300 bp purified from salmon sperm nuclei are shown after incubation with increasing amounts of ISWI, in the presence or absence of PARP and in the presence or absence of PARP plus its competitive inhibitor 3-AB. 125 ng of a mixed length DNA were incubated with 2, 4, 8, and 16 nmol of ISWI, 0.4 nmol of PARP, and 5 mM 3-AB. In the absence of PARP, a mass excess of ∼8 fold ISWI/DNA is sufficient to start to shift the bulk of the DNA population. In contrast, when PARP is included in the bandshift reaction, the mass excess of ISWI necessary to shift the DNA population to the same extent is at least 2-fold greater (16-fold). The reduction in DNA affinity is reversed in the presence of 3-AB.
(1.06 MB TIF)

Figure S4 Effect of Free PAR on the ATPase Activity of ISWI (A) Known amounts of free poly-ADP-ribose [Standard] (Alexis Biochemicals) are compared, by Immuno-dot, with serial dilutions of total PAR—free and covalently attached—produced in standard ATPase/PARylation dual assay reactions in the presence of DNA [DNA] or UV-treated chromatin [CHR] (4 nmol ISWI, 0.03 nmol PARP, 1 μg of activated DNA, or 100 ng in vitro assembled UV-treated chromatin). The amount of PAR produced by PARP, in the presence of DNA or UV-treated chromatin was estimated by quantification of Western blot with aPAR antibody.
(B) ATP hydrolysis of recombinant ISWI incubated with activating DNA in the presence of up to 100 pmol poly-ADP-ribose (∼5× excess the amount produced in ATPase/PARylation dual assay in the presence of DNA).
(C) ATP hydrolysis of recombinant ISWI incubated with UV-treated chromatin in the presence of up to 50 pmol of poly-ADP-ribose (∼5× excess the amount produced in ATPase/PARylation dual assay in the presence of chromatin). In conclusion, a 5-fold excess of free PAR over the amount produced in a standard ATPase/PARylation reaction does not inhibit ISWI DNA-dependent or chromatin-stimulated ATPase activity.
(344 KB TIF)

Figure S5 Mobility Shift Assays Scheme Schematic representation of the temporal order in which we added ISWI, polynucleosomes [Poly], mononucleosomes [Mono], PARP, activating DNA, and 3-aminobenzamide [3-AB] in the mobility shift assays presented (A) in Figure 6A and 6B and (B) in Figure 6C and 6D.
(141 KB TIF)

Figure S6 The *Parp^C03256^* Mutant Fails to Respond to Heat Shock Immuno-FISH using an aPAR antibody (green) and a DNA probe for the *hsp70* loci at 87A and 87C (red), was carried out on polytene chromosomes prepared from *Parp^C03256^* third instar larvae maintained under (A) non–heat-shock or (B) under heat-shock conditions. The data are presented as a merge chromosome image. Chromosomes were also stained with DAPI to visualize DNA (blue). The cytological *hsp70* loci 87A and 87C, are indicated by arrows. The *Parp^C03256^* mutant fails to respond to heat-shock stimuli since no puffing and no hyper-PARylation at 87A and 87C loci is observed after heat-shock. The residual PARP activity detected by the aPAR antibody is comparable to the one presented in Fig 7A.
Anti-ISWI immunostaining (green) and in situ hybridization for the *hsp70* loci 87A and 87C (red) are also compared, as a “split” chromosome image, (C) before or (D) after heat shock. Arrowhead indicates ISWI binding at the 87A *hsp70* locus. (-) non heat-shock and (hs) heat-shock conditions. ISWI remains bound to the 87A locus before and after heat-shock in the *Parp^C03256^* mutant, suggesting that ISWI binding at 87A locus is directly regulated by the activity of PARP.
(381 KB TIF)

## Abbreviations

3-AB: 3-aminobenzammide
GFP: green fluorescent protein
PARG: poly-ADP-ribose glycohydrolase
PARP: poly-ADP-ribose polymerase
PARylated: poly-ADP-ribosylated
TAP: tandem affinity purification
